# Nutraceuticals Targeting Cannabinoid Receptor 1 and Transient Receptor Potential Vanilloid 1 for Pain Relief: A Computational Screening Approach

**DOI:** 10.7759/cureus.81807

**Published:** 2025-04-06

**Authors:** Tahseen Hasan, Mostafa Mohammadi, Ali Jabbari, Hamed A Flaifel, Hassan Mirzaei

**Affiliations:** 1 Department of Anesthesiology, Ischemic Disorders Research Center, Golestan University of Medical Sciences, Gorgan, IRN; 2 Department of Anesthesiology and Intensive Care, Imam Khomeini Hospital Complex, Tehran University of Medical Sciences, Tehran, IRN; 3 Department of Anesthesiology and Critical Care Medicine, Ischemic Disorders Research Center, Golestan University of Medical Sciences, Gorgan, IRN; 4 Department of Anesthesia Techniques, Al-Farqadin University College, Basra, IRQ; 5 Department of Integrative or Complementary Medicine, Ischemic Disorders Research Center, Golestan University of Medical Sciences, Gorgan, IRN

**Keywords:** admet, drug-likeness, molecular docking, molecular dynamics simulation, mu-opioid receptor, natural compounds, trpv1

## Abstract

Objective: This study examined the binding affinities and therapeutic potential of natural products targeting pain-related receptors using molecular docking and molecular dynamics (MD) simulations. Drug-like properties and absorption, distribution, metabolism, excretion, and toxicity (ADMET) analyses were also conducted.

Methods: AutoDock Vina (The Scripps Research Institute, La Jolla, CA, USA) was used for docking against pain-related receptors, including transient receptor potential vanilloid 1 (TRPV1), cyclooxygenase-2 (COX-2), cannabinoid receptor 1 (CB1), mu-opioid receptor, and nicotinic acetylcholine receptors. Celecoxib was included as a reference drug for docking score comparison. Protein-ligand complex stability was assessed via 100-nanosecond (ns) MD simulations using GROMACS (GROningen MAchine for Chemical Simulations; the University of Groningen, Netherlands), analyzing root mean square deviation (RMSD) and radius of gyration (Rg). Drug-likeness was evaluated by Lipinski’s rule of five, and ADMET analysis was performed for pharmacokinetics and toxicity profiling.

Results: Ginsenoside Rb1 exhibited a strong affinity for TRPV1 (-9.5 kcal/mol) and mu-opioid (-9.0 kcal/mol) receptors, suggesting its potential as a non-opioid analgesic candidate. Cyanidin 3-O-rutinoside demonstrated high binding to TRPV1 (-9.35 kcal/mol), COX-2 (-9.65 kcal/mol), and CB1 (-9.18 kcal/mol), surpassing the reference drug celecoxib (-7.22 kcal/mol) in COX-2 binding. MD simulations confirmed complex stability, with RMSD (~3.0 Å) and Rg (~3.0 nm) values lower than unbound proteins. Most compounds met Lipinski’s criteria, indicating good oral bioavailability. ADMET analysis revealed favorable absorption and distribution with low toxicity.

Conclusion: Ginsenoside Rb1 and cyanidin 3-O-rutinoside exhibit high binding affinity, stability, and favorable pharmacokinetic properties, supporting their potential as non-opioid analgesic candidates. Their ability to modulate pain pathways in vitro and in vivo warrants further investigation.

## Introduction

Chronic pain affects millions worldwide, presenting both substantial health and financial challenges. Despite serious risks of dependency, tolerance, and addiction, opioids remain the primary treatment option. This underscores the urgent need for safer and more effective pain management strategies [1]. Natural compounds derived from phytochemicals and secondary metabolites exhibit anti-inflammatory and analgesic properties, contributing to pharmaceutical and cosmeceutical advancements. Advanced drug delivery systems integrate herbal medicine extracts with established pain-relieving effects to enhance targeted pain management [2].

While natural compounds offer pain relief, concerns regarding the safety and addiction potential of opioid derivatives have intensified the search for alternative therapeutic approaches. Traditional Chinese medicine, including acupuncture, has been studied for its analgesic benefits [3]. Additionally, multimodal analgesic therapies incorporating natural compounds are being explored in both human and veterinary medicine [4,5].

Three bioactive agents - ginsenoside Rb1, curcumin, and cyanidin 3-O-rutinoside - demonstrate promise in modulating pain-related and inflammatory molecular signaling pathways [6]. Pain management relies on targeting key receptors such as transient receptor potential vanilloid 1 (TRPV1), cyclooxygenase-2 (COX-2), cannabinoid receptor 1 (CB1), mu-opioid, and nicotinic acetylcholine receptors, which are central to pain perception. Natural compounds interacting with these receptors may reduce opioid dependence.

Molecular docking technologies predict protein-small molecule binding patterns, facilitating the discovery of novel pain modulators with safer pharmacological profiles. This study evaluates the binding interactions and stability of selected natural compounds through molecular modeling and absorption, distribution, metabolism, excretion, and toxicity (ADMET) analysis, aiming to develop opioid-free pain management alternatives.

## Materials and methods

Molecular docking

AutoDock Vina (The Scripps Research Institute, La Jolla, CA, USA) was used to estimate binding affinities and interactions between the selected natural compounds and pain-related receptors, including TRPV1, COX-2, CB1, mu-opioid, and nicotinic acetylcholine receptors. Celecoxib was used as a reference compound. The grid box dimensions for docking were set to 40 × 40 × 40 Å with a spacing of 0.375 Å to fully encompass the ligand-binding region. Docking was performed using an exhaustiveness value of 8 to optimize accuracy. The best docking poses were selected based on binding energy scores and key interactions with active-site residues, assessed using MAESTRO software (Schrödinger, LLC, New York, USA) for visualization [7].

Protein preparation

Protein structures retrieved from the Protein Data Bank (PDB) served as templates for conducting molecular docking experiments. The PDB IDs of TRPV1 (3J5P) and COX-2 (5IKR), along with CB1 (6KPF), mu-opioid receptors (8EF5) and nicotinic acetylcholine receptor (7KOQ) served as the templates for structural investigations. The docking structures underwent detailed preparation before docking by implementing AutoDockTools to remove water molecules while attaching polar hydrogen atoms and assigning Gasteiger partial charges [8].

Ligand selection

A total of 550 phytochemicals with reported pain-relief potential were screened as candidate ligands. Compounds such as curcumin, capsaicin, gingerol, ginsenoside Rb1, and cyanidin 3-O-rutinoside were selected based on their ability to modulate pain pathways. Curcumin reduces inflammatory markers tumor necrosis factor-alpha (TNF-α) and nitric oxide. Through its binding to TRPV-1, the pain-relieving compound capsaicin in chili peppers functions as a neuropathic pain treatment by controlling brain signals related to pain. The TRPV-1 receptor agonist effect of gingerol located in ginger shows potential for treating patients with inflammatory and neuropathic pain disorders. Research findings confirm that natural compounds fit well within scientific evidence showing that phytochemicals offer dependable pain relief strategies [9-11].

Ligand preparation

The 3D structures of selected natural compounds and the reference drugs were downloaded from the PubChem Database. At first, these structures were available only in SDF format, which was converted into PDB format using software named OpenBabel (OpenBabel Project). All the ligands were energy minimised by using the force field MMFF94, and Gasteiger charge assignment was given to the ligands for effective docking [7].

Drug-likeness analysis

The SWISSADME tool (Swiss Institute of Bioinformatics (SIB), Lausanne, Switzerland) assessed drug-likeness based on Lipinski’s rule of five, evaluating molecular weight, hydrogen bond donors (HBDs)/acceptors (HBAs), and the octanol/water partition coefficient. Compounds satisfying these criteria were considered promising for further therapeutic development.

ADMET prediction

The SWISSADME tool was also used for ADMET predictions. Key parameters included blood-brain barrier (BBB) permeability, human intestinal absorption (HIA), cytochrome P450 inhibition, and toxicity risks (mutagenicity and hepatotoxicity). These predictions helped assess the pharmacokinetic behavior and safety profile of the selected compounds [12].

Molecular dynamics (MD) simulation

MD simulations were performed using GROMACS 2020 (University of Groningen, Netherlands) with the CHARMM27 force field [13]. SWISSPARAM generated topology files for ligands. A 100-nanosecond (ns) simulation was chosen based on prior studies for stability assessment. Energy minimization was conducted using the Amber 96 force field, followed by two equilibration steps: 100 picoseconds (ps) under NVT (constant volume and temperature) and 100 ps under NPT (constant pressure and temperature) at 300 K and 1 bar. The system was neutralized with seven chloride ions in a TIP3P water model. A cubic simulation box containing 36,250 water molecules was used, and Particle Mesh Ewald (PME) was applied for long-range electrostatics. Trajectory analysis, including root mean square deviation (RMSD) and radius of gyration (Rg) calculations, was conducted using QtGrace (University of Texas, USA) and VMD (University of Illinois, USA).

## Results

Molecular docking

The binding affinity of selected compounds was evaluated against five target proteins using AutoDock Vina: TRPV1 (PDB ID: 3J5P), COX-2 (PDB ID: 5IKR), CB (PDB ID: 6KPF), mu-opioid receptor (PDB ID: 8EF5), and nicotinic acetylcholine receptor (PDB ID: 7KOQ). Several natural compounds demonstrated the potential to interact with multiple pain-related targets (Table 1).

**Table 1:** Results of the molecular docking study. PDB: Protein Data Bank

Compounds	PDB (kcal/mol)				
	3J5P	5KIR	6KPF	8EF5	7KOQ
Ginsenoside Rb1	-10.46	-6.57	-7.04	-11.94	-5.49
Prodelphinidin B3	-9.54	-5.36	-5.50	-8.42	-2.91
Oleuropeinyl monoglucoside	-9.49	-9.00	-9.00	-8.85	-3.70
Cyanidin 3-O-rutinoside	-9.35	-9.62	-9.18	-8.47	-8.17
Prostacyclin-PG12	-8.98	-9.66	0.00	-7.97	-4.64
Rutin	-8.36	-8.90	-9.31	-7.65	-7.14
Curcumin	-8.07	-9.01	-9.01	-8.55	-5.51
Procyanidin A2	-7.60	-5.40	-6.86	-7.24	-3.62
Ginkgolide-A	-7.59	-6.38	-7.40	-7.39	-6.79
Bis-coumarin	-7.56	-9.36	-7.93	-7.83	-5.30
Phenolic acid	-7.55	-7.34	-8.14	-7.17	-2.57
Rosmarinic acid	-7.37	-8.33	-8.41	-8.35	-5.21
Chrysanthemin	-7.03	-9.16	-8.28	-7.33	-3.64
Chlorogenic acid	-6.97	-6.93	-6.71	-6.11	-3.61
Capcicin	-6.88	-7.64	-7.19	-6.91	-4.90
Kaempferide 3-glucoside	-6.85	-8.52	-7.54	-6.80	-3.93
Quercetin 3-O-glucopyranoside	-6.82	-8.40	-8.43	-7.15	-2.90
Piperine	-6.48	-7.15	-7.08	-6.92	-4.28
Celecoxib	-7.21	-9.33	-7.93	-7.22	-4.92

TRPV1 (PDB ID: 3J5P)

**Figure 1:**
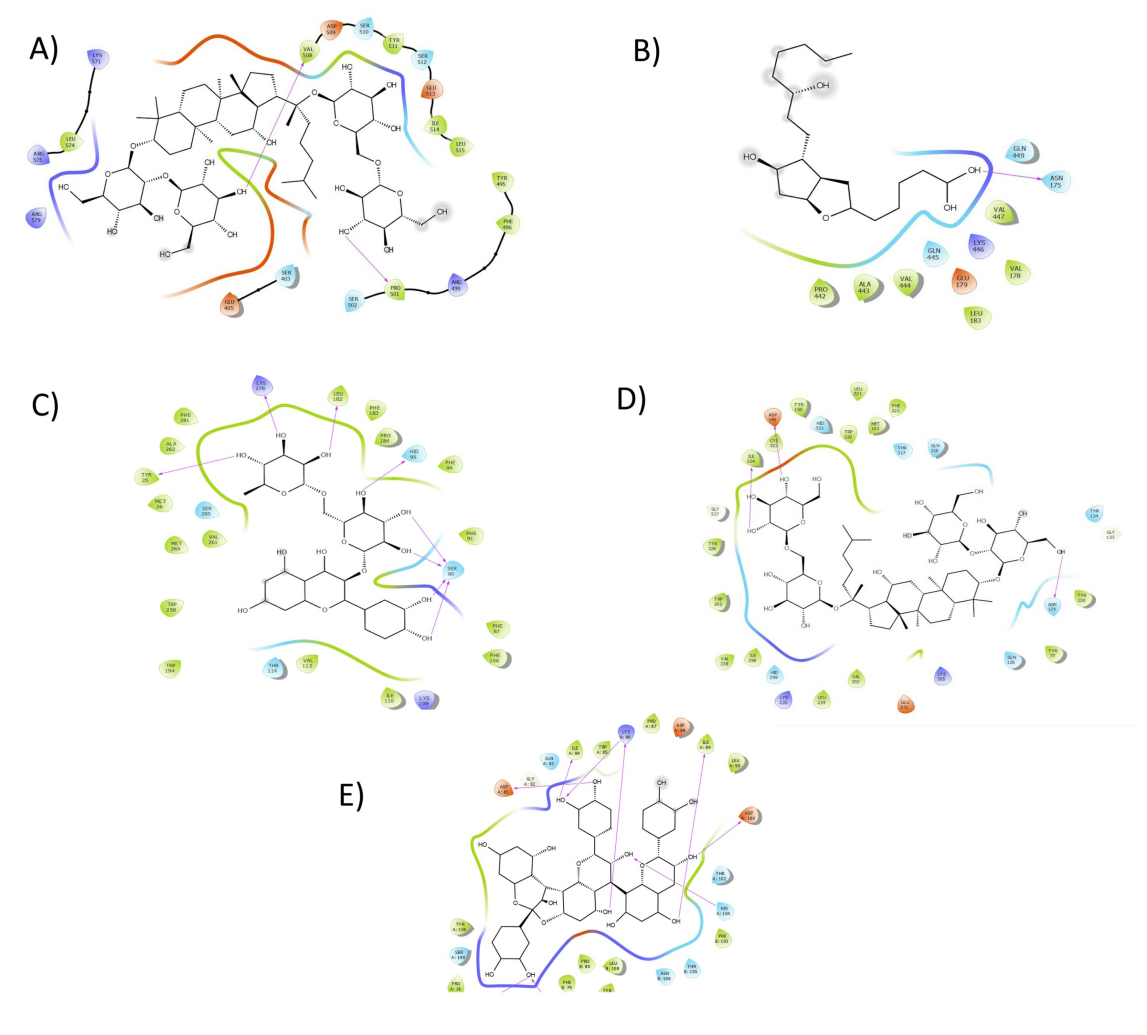
Presentation of 2D model of interactions between (A) receptor of TRPV1 (PDB:3J5P) with ginsenoside Rb1; (B) receptor of COX-2 (PDB: 5IKR) with cyanidin 3-O-rutinoside; (C) cannabinoid receptor 1 (PDB: 6KPF) with rutin; (D) mu-opioid receptor (PDB ID: 8EF5) with ginsenoside Rb1; (E) nicotinic acetylcholine receptor (PDB ID: 7KOQ) with cyanidin 3-O-rutinoside. TRPV1: transient receptor potential vanilloid 1; COX-2: cyclooxygenase-2; PDB: Protein Data Bank

Among tested compounds, ginsenoside Rb1 showed the strongest binding (-10.46 kcal/mol), followed by prodelphinidin B3 (-9.54 kcal/mol) and oleuropeinyl monoglucoside (-9.49 kcal/mol). Ginsenoside Rb1 interacted with key active site residues through both hydrophobic (Leu574, Val508, Tyr511, Ile514, Leu511, Tyr495, Phe496, Pro501) and polar interactions (Ser510, Ser512, Asp509, Glu513, Arg499, Ser502, Ser403, Arg579, Arg575, Glu405, Lys571), forming four strong hydrogen bonds (1.71-2.99 Å, see Figure 1A and Table 2). Prodelphinidin B3 and oleuropeinyl monoglucoside also suggest potential as TRPV1 modulators, possibly blocking the receptor in an inactive state. Curcumin (-7.2 kcal/mol) and the non-steroidal anti-inflammatory drug (NSAID) reference drug celecoxib (-7.2 kcal/mol) further support the dual approach of receptor modulation and anti-inflammatory action.

**Table 2:** Key amino acid residues between selected compound and targets.

Bonding type/targets	3J5R	5IKR	6KPF	7KOQ	8EF5
Hydrophilic	Leu574, Val5088, Tyr511, Ile514, Leu5115, Tyr495, Phe496, Pro501,	Val447, Val178, Leu183, Val444, Pro442, Ala443	Phe281, Ala282, Tyr25, Met26, Met265, Val261, Leu182, Phe183, Pro184, Trp258, Phe94, Phe87, Phe107, Ile110, Val113, Trp194	Ile84, Trp85, Pro87, Ile89, Leu90, Phe103, Leu108, Pro80, Phe79, Tyr117, Tyr7, Met57, Val77, Tyr150, Pro16, Leu17	Tyr150, Cys323, Ile324, Trp320, Leu221, Phe223, Met153, Tyr328, Trp295, Val238, Ile298, Val302, Tyr130, Tyr77
Polar	Ser510, Ser512, Asp509, Glu513, Arg499, Ser502, Ser403, Arg579, Arg575, Glu405, Lys571	Gln449, Asn175, Lys446, Glu179	Thr114, Lys109, Ser90, His95, Ser285, Lys278	Gln83, Asp81, SEr149, Lys86, Asp88, Asp100, Thr102, His104, Thr105, Arg78, Glu18	Lys305, Gln126, Asn129, Thr134, Glu231, Lys235, His299, Asp149, His321, Tyr317, Gln316
Hydrogenous	Val508, Ser502, Pro501	Gln440, Asn175,	Ser285, Ala282, Lys278, Tyr25, Ser90, Leu182, His95	Lys86, His104, Tyr7, Arg78, Asp100, Ile89, Ile84, Leu17, Lys86, Asp81, Asp88	Lys305, Asn129, Ile77, Leu234, Thr220

COX-2 (PDB ID: 5IKR)

Quercetin 3-O-rutinoside (-9.62 kcal/mol) and cyanidin 3-O-rutinoside (-9.01 kcal/mol) displayed strong binding with COX-2. Hydrophobic interactions involved residues like Val447, Val178, Leu183, Val444, Pro442, and Ala443, while polar contacts were seen with Gln449, Asn175, Lys446, and Glu179. Cyanidin 3-O-rutinoside formed two hydrogen bonds (2.3-2.7 Å) with Gln440 and interacted with Asn175 (Figure 1B). Additionally, prostacyclin-PG12 (-8.2 kcal/mol), curcumin (-7.0 kcal/mol), and oleuropeinyl monoglucoside also showed significant affinities. For comparison, the COX-2 inhibitor celecoxib had a binding affinity of -9.6 kcal/mol.

Cannabinoid Receptor (CB) (PDB ID: 6KPF)

Rutin achieved the best binding energy (-9.31 kcal/mol) with the CB, followed by oleuropeinyl monoglucoside (-9.00 kcal/mol). Rutin established 10 hydrogen bonds with residues such as Ser285, Ala282, Lys278, and Tyr25 and engaged in hydrophobic interactions with several key residues (Figure 1C). Curcumin (-7.0 kcal/mol) also showed promising binding, supporting its analgesic and anti-inflammatory role. Overall, rutin and cyanidin 3-O-rutinoside demonstrated the highest affinities among active compounds.

Mu-Opioid Receptor (PDB ID: 8EF5)

Ginsenoside Rb1 exhibited the strongest binding to the mu-opioid receptor (-11.94 kcal/mol), suggesting potent pain modulation via this pathway. Prodelphinidin B3 (-8.42 kcal/mol), oleuropeinyl monoglucoside (-8.85 kcal/mol), and cyanidin 3-O-rutinoside (-8.47 kcal/mol) also showed good binding. Prostacyclin-PG12 recorded -8.0 kcal/mol, while curcumin had a moderate affinity (-7.2 kcal/mol). For context, reference opioids fentanyl (-7.6 kcal/mol) and morphine (-10.9 kcal/mol) interact with the receptor through a mix of hydrophobic interactions (with residues such as Tyr150, Cys323, Ile324, etc.) and hydrogen bonds (with Lys305, Asn129, etc.) (see Figure 1D).

Nicotinic Acetylcholine Receptor (PDB ID: 7KOQ)

Cyanidin 3-O-rutinoside showed strong binding (-8.2 kcal/mol) with the nicotinic acetylcholine receptor, interacting with hydrophobic residues (Ile84, Trp85, Pro87, etc.) and forming 13 hydrogen bonds (1.46-2.82 Å, Figure 1E). Rutin also exhibited a notable binding affinity (-7.1 kcal/mol). The reference compound mivacurium, acting as a neuromuscular blocker, had a binding energy of -7.9 kcal/mol.

MD simulation

**Figure 2:**
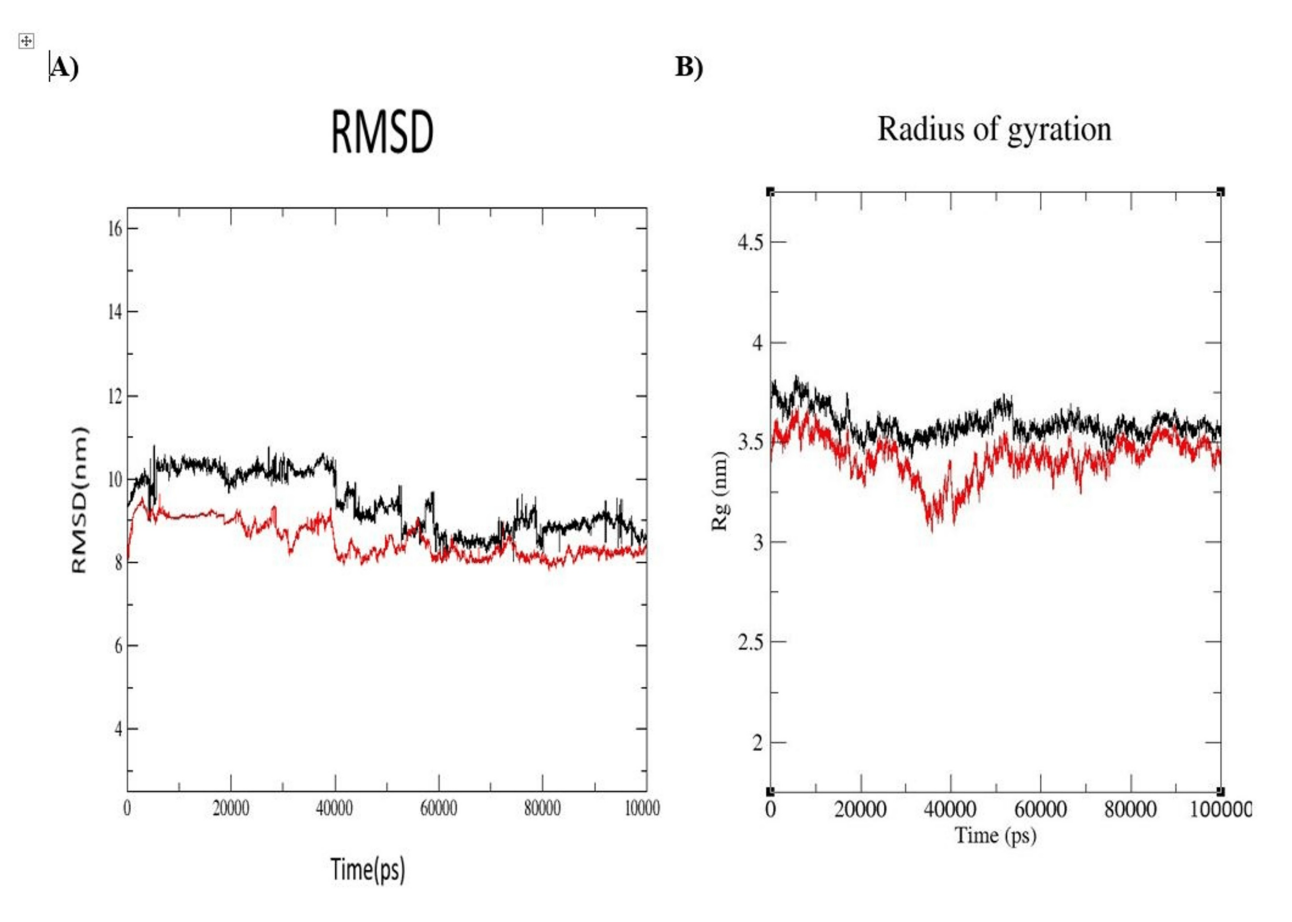
(A) RMSD plots of the 3J5P protein (black) and its complex with cyanidin 3-O-rutinoside (red) in water at 300 K over a 100 ns MD simulation. (B) Radius of gyration (Rg) analysis of the 3J5P protein (black) and its complex with cyanidin 3-O-rutinoside (red) in water during the 100 ns MD simulation. RMSD: root mean square deviation; MD: molecular dynamics

The RMSD of the protein alone and the protein-ligand complex was recorded throughout the 100 ns MD simulation of the protein (Figure 2A). Namely, the stability of the protein alone can be observed where the RMSD average remains stable at 3.5 Å already after 10 ns of the simulation. Likewise, in the protein-ligand complex, a stable RMSD is achieved after 15 ns with an RMSD of about 3.0 Å. The observation of lower RMSD in the overall protein-ligand complex as compared to the protein only indicates that the ligand helps in the stabilization of the protein during simulation. The Rg plot (Figure 2B) for the protein alone (black) and in complex with the ligand (red) gives information about how compact the protein is overall during the simulation. For the protein alone, the Rg oscillates around 3.5 nm, and there is a decrease in the first 10 ns, indicating a sign of structural relaxation.

**Figure 3:**
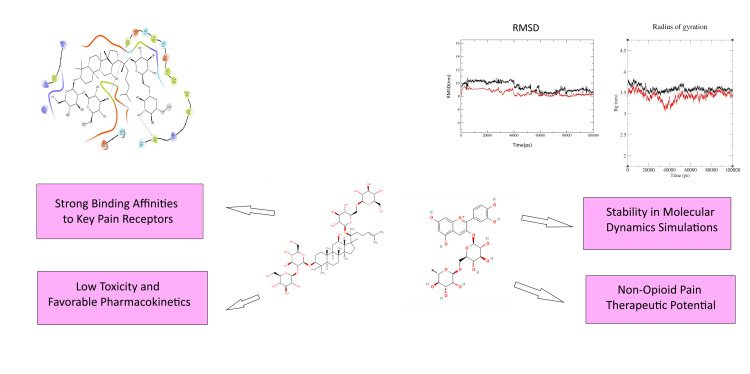
Graphical abstract. RMSD: root mean square deviation

Overall, the protein-ligand complex appears to be denser, with the Rg being relatively below 3.5 nm. The reduction of the Rg value reveals that the average distance is smaller than that of the protein alone towards the presence of the ligand, which generally leads to a more compact and stable conformation of the protein (Figure 3).

Drug-likeness properties

The drug-likeness properties of the compounds (Table 3) were assessed using Lipinski’s rule of five, which estimates the likelihood of these compounds to be orally active in humans. A list of evaluation criteria was considered, which included the molecular weight, HBDs and HBAs, log P, and the total number of rotatable bonds (NRB). The molecular weight of ginsenoside Rb1 was presented at 1101.25, which was over the threshold, and it suggests that the molecule may face problems with oral absorption. It also provided high values for HBD and HBA, indicating that it cannot have good permeability.

**Table 3:** Drug-likeness profiles of selected compounds. MW: molecular weight; log S: logarithm of water solubility; log P: logarithm of compound partition coefficient between octanol and water; HBA: number of hydrogen bonds acceptors; HBD: number of hydrogen bond donors; NRB: number of rotatable bonds; TPSA: topological polar surface

Compound	MW	Log S	C log P	HBA	HBD	NRB	TPSA
Ref.	-	≥4	≤5	≤10	≤5	≤10	<140
Ginsenoside Rb1	1109	-3	-1.6	23	15	16	377
Prodelphinidin B3	610	-6.3	1.7	14	12	3	261
Oleuropeinyl monoglucoside	702	-4.3	-2.3	18	9	14	281
Cyanidin 3-O-rutinoside	595	-5.7	-0.4	14	10	6	240
Prostacyclin-PGI2	704	-6.2	-0.7	10	6	20	174
Rutin	610	-2.2	-0.87	16	10	6	266
Curcumin	368	-4.8	3.6	6	2	8	93
Procyanidin A2	576	-0.7	2.4	12	9	2	210
Ginkgolide-A	408	-2.1	0.6	9	2	1	129
Bis-coumarin	336	-3.7	1.94	6	2	2	93
Rosmarinic acid	360	-4.0	3.0	8	5	7	145
Chrysanthemin	484	-5.7	-0.9	11	8	4	181
Chlorogenic acid	354	-2.0	-0.27	9	6	5	165
Capsaicin	305	-3.0	3.75	3	2	9	58
Kaempferide 3-glucoside	462	-2.1	1.1	11	6	5	175
Quercetin 3-O-glucopyranoside	464	-3.0	0.4	12	4	4	207
Piperine	285	-3.3	2.78	3	0	3	38
Celecoxib	381	-4.9	4.01	7	1	3	86

Flavonoids, prodelphinidin B3, and cyanidin 3-O-rutinoside were also chosen as they have codes greater than the molecular weight of 500 Da, which are/are 612.52 Da and 594.51 Da, respectively. However, these compounds exhibited relatively low logP values, which indicated slightly improved lipophilicity than that of ginsenoside Rb1. Both curcumin and celecoxib fell within Lipinski’s set of rules for desired drug-like properties, implying a good drug-like character. Celecoxib more so correlated very well with the criteria for passing lipophilicity (Table 3). Most of the compounds conformed to Lipinski’s rule of five and presented a drug-like character, so mild structural change may improve their characteristics.

ADMET analysis

ADMET analysis indicated that most compounds have favorable absorption, distribution, and metabolic profiles. Ginsenoside Rb1 and cyanidin 3-O-rutinoside, for instance, showed promising ADMET properties (Table 4). Curcumin demonstrated excellent absorption but low bioavailability due to rapid metabolism and poor solubility, underscoring the need for improved formulation strategies. While ginsenoside Rb1 appears effective for neuropathic pain, its low BBB penetration suggests it may be more suited for peripheral pain management.

**Table 4:** ADMET properties of selected compounds. ADMET: absorption, distribution, metabolism, excretion, and toxicity; BBB: blood brain barrier; HIA: human intestinal absorption, Caco-2: a model of the intestinal epithelial barrier; P-GI: P-glycoprotein inhibitor; AMES: Salmonella typhimurium reverse mutation assay; CIG: carcinogens; HPT: hepatotoxicity; AOC: acute oral toxicity

Compound	BBB	HIA	Caco2	P-GI	CYP450-2C9	CYP450-2D6	CYP450-3A4	AMES	CIG	HPT	AOC
Ref.	-	-	-	No	No	No	No	No	No	No	-
Ginsenoside Rb1	0.57	0.61	0.90	0.72	0.86	0.93	0.95	0.93	0.96	0.93	4.02
Prodelphinidin B3	0.52	0.64	0.88	0.69	0.88	0.91	0.62	0.83	0.94	0.95	4.20
Oleuropeinyl monoglucoside	0.48	0.62	0.87	0.67	0.84	0.89	0.75	0.86	0.93	0.92	4.31
Cyanidin 3-O- rutinoside	0.77	0.77	0.91	0.65	0.90	0.86	0.92	0.53	0.67	0.67	2.3
Prostacyclin-PGI2	0.78	0.62	0.93	0.62	0.92	0.88	0.93	0.54	0.94	0.91	4.3
Rutin	0.85	0.80	0.91	0.87	0.90	0.95	0.92	0.51	0.96	0.96	2.5
Curcumin	0.83	0.85	0.93	0.89	0.91	0.94	0.89	0.62	0.95	0.94	3.4
Procyanidin A2	0.65	0.64	0.87	0.66	0.85	0.91	0.74	0.80	0.96	0.92	3.1
Ginkgolide-A	0.83	0.92	0.66	0.64	0.90	0.96	0.90	0.50	0.91	0.92	2.88
Bs-coumarin	0.83	0.87	0.58	0.93	0.89	0.96	0.90	0.90	0.95	0.94	3.1
Rosmarinic acid	0.57	0.67	0.84	0.74	0.86	0.91	0.93	0.86	0.84	0.91	2.7
Chrysanthemin	0.56	0.61	0.82	0.58	0.89	0.92	0.86	0.91	0.88	0.93	3.3
Chlorogenic acid	0.84	0.84	0.92	0.68	0.91	0.88	0.92	0.93	0.95	0.92	2.9
Capsaicin	0.55	0.81	0.64	0.87	0.91	0.87	0.93	0.91	0.94	0.88	3.3
Kaempferide 3-glucoside	0.68	0.78	0.89	0.91	0.93	0.94	0.95	0.86	0.93	0.92	2.9
Quercetin 3-O-glucopyranoside	0.67	0.78	0.86	0.79	0.91	0.93	0.93	0.91	0.93	0.88	3.3
Piperine	0.58	0.89	0.84	0.77	0.91	0.87	0.92	0.94	0.88	0.93	3.6
Celecoxib	0.97	1.0	0.88	0.86	0.61	0.85	0.79	0.71	0.79	0.84	2.4

## Discussion

Molecular docking

This study employed molecular docking to evaluate five natural compounds - ginsenoside Rb1, curcumin, rutin, cyanidin 3-O-rutinoside, and prostacyclin-PG12 - as potential non-opioid analgesics by assessing their interactions with molecular targets involved in pain.

Ginsenoside Rb1 demonstrated the highest binding affinity (-9.5 kcal/mol) to the TRPV1 receptor, a key mediator of pain sensation [14]. This suggests that ginsenoside Rb1 could inhibit receptor activation and block pain signaling, supporting its potential as a TRPV1-targeted drug [15,16].

Regarding COX-2 inhibition (PDB ID: 5IKR), cyanidin 3-O-rutinoside (-9.62 kcal/mol) and curcumin (-9.01 kcal/mol) exhibited strong binding, reinforcing their role in reducing prostaglandin-mediated inflammation [17]. Hydrophobic and polar interactions, particularly with Val447 and Gln449, indicate a strong inhibitory effect, aligning with research on COX-2 inhibitors reducing opioid use post-surgery [9]. Prostacyclin-PG12 also showed notable COX-2 binding (-8.2 kcal/mol), indicating anti-inflammatory potential.

Curcumin's efficacy (-7.0 kcal/mol) in COX-2 inhibition supports its known anti-inflammatory role [18,19]. These findings suggest that these compounds might offer NSAID-like benefits with fewer side effects. Rutin demonstrated moderate CB1 receptor binding (-7.5 kcal/mol), implicating it in endocannabinoid-mediated analgesia [20]. Given its potential to modulate CB1, rutin warrants further study as an opioid alternative. Curcumin’s moderate binding affinity to CB1 further supports its multi-targeted analgesic properties.

Ginsenoside Rb1 also displayed high binding affinity (-9.0 kcal/mol) to the mu-opioid receptor, suggesting opioid-like analgesic effects with reduced dependency risks [21]. Curcumin exhibited moderate binding (-7.0 kcal/mol), supporting its potential as an adjunct to opioid analgesics [22]. Cyanidin 3-O-rutinoside’s hydroxyl and aromatic structures enhance receptor interactions, making it a viable pain mediator. Its glycosidic linkage further improves solubility and bioavailability, supporting its role in analgesia.

Fluorescent studies on cyanidin 3-O-rutinoside and curcumin suggest interactions with nicotinic acetylcholine receptors, potentially disrupting pain pathways [23]. These findings highlight an alternative pain management approach.

Ginsenoside Rb1’s binding to TRPV1 and mu-opioid receptors indicates dual pain inhibition, stabilizing receptor conformation, and mimicking endogenous opioids. Curcumin’s interactions with COX-2 and CB1 suggest a dual mechanism, reducing inflammation and enhancing endocannabinoid signaling. Rutin’s CB1 modulation presents a novel analgesic pathway.

These results align with prior research on natural analgesics. Ginsenoside Rb1’s TRPV1 and mu-opioid affinities support its role in neuropathic pain relief [15,16]. Curcumin’s COX-2 and CB1 interactions reinforce its anti-inflammatory and analgesic properties [24,25]. The findings suggest further exploration of these compounds for pain management.

Molecular docking suggests natural compounds as potential analgesics targeting pain-related pathways [26]. However, functional assays and clinical studies are necessary to validate their efficacy.

MD simulation

RMSD analysis confirmed the structural stability of both apo-protein and protein-ligand complexes during 100 ns of MD simulation. The protein alone stabilized slightly earlier but showed higher fluctuations. Lower RMSD values for the protein-ligand complex suggest ligand-induced conformational stability, consistent with prior findings [27].

The Rg analysis further supported this conclusion. Protein-ligand complexes displayed lower Rg values than apo-proteins, indicating ligand-induced structural compaction. Similar trends in the literature suggest ligand binding enhances protein stability [27,28]. These findings highlight the role of ligand binding in stabilizing target proteins, reinforcing their potential as therapeutic agents.

Drug-likeness properties and ADMET prediction

The drug-likeness evaluation indicated moderate oral bioavailability for these compounds. Ginsenoside Rb1 exhibited strong binding affinities but poor permeability due to its high molecular weight and hydrogen bond donor/acceptor values. Conversely, curcumin and celecoxib complied with Lipinski’s rules, suggesting better bioavailability.

Pharmacokinetic analysis from ADMET studies revealed good absorption and low toxicity for most compounds, particularly curcumin and rutin, with minimal cytochrome P450 interactions, supporting their safety as analgesics. Simoben et al. noted that natural compounds often struggle with oral bioavailability despite strong activity. Ginsenoside Rb1’s high molecular weight (400.25 Da) suggests poor absorption, while curcumin and celecoxib exhibit favorable oral bioavailability [29].

Celecoxib’s well-documented COX-2 inhibition, combined with good molecular weight and lipophilicity, supports its analgesic potential. Prodelphinidin B3 and cyanidin 3-O-rutinoside also satisfied Lipinski’s rule, indicating potential oral activity despite lower binding affinities [29].

Most compounds demonstrated satisfactory pharmacokinetic profiles, but curcumin’s low bioavailability remains a challenge. Strategies like nanoparticle delivery or structural modifications may improve its clinical efficacy [30]. Silico studies suggest that ginsenoside Rb1, curcumin, and rutin are promising non-opioid analgesic candidates. Their broad target activity and low toxicity warrant further in vitro and in vivo exploration to validate their therapeutic potential.

While this study provides valuable insights into the potential of natural compounds as analgesics, it has certain limitations. Molecular docking and MD simulations offer information about binding interactions and stability in theoretical scenarios but do not fully represent biological systems under real conditions. The study lacks extensive examination of key factors such as metabolic stability, bioavailability, and possible secondary effects. Laboratory and animal studies are necessary to validate therapeutic potential, as experimental testing provides crucial confirmation of drug efficacy. Additionally, clinical translation presents challenges, particularly regarding the bioavailability of compounds like curcumin and ginsenoside Rb1, necessitating advanced formulation strategies such as nanoparticle-based delivery systems. Determining optimal dosing for nutraceuticals in clinical settings requires comprehensive pharmacokinetic and pharmacodynamic studies, while safety, metabolism, and potential drug interactions must be thoroughly evaluated for regulatory approval. Future research should focus on these aspects to bridge the gap between computational predictions and clinical applications.

## Conclusions

This study explored the potential of natural compounds as pain relievers using molecular docking and MD simulations. Our findings indicate that ginsenoside Rb1 and curcumin emerge as promising candidates, exhibiting strong binding affinities to key pain-related receptors. Cyanidin 3-O-rutinoside demonstrated enhanced binding capabilities due to its hydroxyl groups, aromatic rings, and glycosidic linkages. MD simulations over 100 ns confirmed the structural stability of ginsenoside Rb1 and cyanidin 3-O-rutinoside, as reflected in their RMSD and Rg values.

These results provide valuable insights into how natural compounds interact with pain receptors, potentially contributing to novel pain management strategies. Our findings align with previous research and highlight the favorable ADMET profiles of these non-opioid alternatives. Ginsenoside Rb1, curcumin, and rutin show promise as foundational compounds for developing innovative analgesics. However, further in vitro and in vivo studies are essential to validate these interactions and assess their therapeutic efficacy in clinical settings.
